# Members of the endocannabinoid system are distinctly regulated in inflammatory bowel disease and colorectal cancer

**DOI:** 10.1038/s41598-019-38865-4

**Published:** 2019-02-20

**Authors:** Magdalena Grill, Christoph Högenauer, Andreas Blesl, Johannes Haybaeck, Nicole Golob-Schwarzl, Nerea Ferreirós, Dominique Thomas, Robert Gurke, Martin Trötzmüller, Harald C. Köfeler, Birgit Gallé, Rudolf Schicho

**Affiliations:** 1 Otto Loewi Research Center, Division of Pharmacology, Medical University of Graz, Graz, Austria; 2 Division of Gastroenterology and Hepatology, Department of Internal Medicine, Medical University of Graz, Graz, Austria; 3 BioTechMed, Graz, Austria; 4 Department of Pathology, Otto von Guericke University, Magdeburg, Germany; 5 Diagnostic and Research Institute of Pathology, Medical University of Graz, Graz, Austria; 6 Department of Pathology, Medical University of Innsbruck, Innsbruck, Austria; 7 Institute of Clinical Pharmacology, Goethe University, Frankfurt/Main, Germany; 8 Core Facility for Mass Spectrometry, Center for Medical Research, Medical University of Graz, Graz, Austria; 9 Omics Center Graz, BioTechMed-Graz, 8010 Graz, Austria; 10 Core Facility Molekularbiologie, Center for Medical Research, Medical University of Graz, Graz, Austria

## Abstract

Preclinical studies have demonstrated that the endocannabinoid system (ECS) plays an important role in the protection against intestinal inflammation and colorectal cancer (CRC); however, human data are scarce. We determined members of the ECS and related components of the ‘endocannabinoidome’ in patients with inflammatory bowel disease (IBD) and CRC, and compared them to control subjects. Anandamide (AEA) and oleoylethanolamide (OEA) were increased in plasma of ulcerative colitis (UC) and Crohn’s disease (CD) patients while 2-arachidonoylglycerol (2-AG) was elevated in patients with CD, but not UC. 2-AG, but not AEA, PEA and OEA, was elevated in CRC patients. Lysophosphatidylinositol (LPI) 18:0 showed higher levels in patients with IBD than in control subjects whereas LPI 20:4 was elevated in both CRC and IBD. Gene expression in intestinal mucosal biopsies revealed different profiles in CD and UC. CD, but not UC patients, showed increased gene expression for the 2-AG synthesizing enzyme diacylglycerol lipase alpha. Transcripts of *CNR1* and *GPR119* were predominantly decreased in CD. Our data show altered plasma levels of endocannabinoids and endocannabinoid-like lipids in IBD and CRC and distinct transcript profiles in UC and CD. We also report alterations for less known components in intestinal inflammation, such as GPR119, OEA and LPI.

## Introduction

Inflammatory bowel disease (IBD) and colorectal cancer (CRC) are severe diseases of the gastrointestinal tract (GI). The two forms of IBD, ulcerative colitis (UC) and Crohn’s disease (CD) have rapidly increased in the past years in Western countries ranging at a prevalence of more than 200 cases per 100,000 inhabitants^1^. Despite introduction of several new therapeutic agents in recent years, current therapeutic options are insufficient for a successful treatment leading to a high rate of disability and intestinal surgery in IBD patients^2,3^ CRC has been classified as the third most common cancer entity and a mayor cause of cancer death since therapeutic options are limited especially in advanced stages of CRC^4^. Standard treatment for IBD includes anti-inflammatory agents, such as aminosalicylic acid as well as immunomodulators, steroids, and biological agents. For CRC, surgical resection and chemotherapy represent the main curative therapies. The gut is an organ with a well-organized endocannabinoid system (ECS)^5^, and several preclinical studies performed in recent years have indicated that cannabinoids are strongly protective against intestinal inflammation^5,6^ and CRC^7,8^. These findings suggest that human IBD and CRC may be amenable to cannabinoid treatment. In the GI tract, the ECS consists of several components that take part in a multitude of physiological events with the aim to restore homeostasis^5,9,10^. Many non-cannabinoid receptors that are modulated by cannabinoids (e.g. peroxisome proliferator-activated receptors (PPARs) and G protein-coupled receptor 55 (GPR55)) and endocannabinoid-like lipids that do not act via cannabinoid receptors (e.g. oleoyl- (OEA) and palmitoylethanolamide (PEA)) are related to the ECS, therefore, the term “endocannabinoidome” has been coined^11^ to unite all these components. This term includes amongst others (i) cannabinoid (CB) and non-CB receptors (responsive to exo- and endocannabinoids), such as cannabinoid receptor 1 and 2 (CB_1_ and CB_2_), GPR55 and GPR119, PPARs, and transient receptor potential cation channel subfamily V member 1 (TRPV1); furthermore (ii) endogenous receptor ligands (endocannabinoids and endocannabinoid-like lipids), such as anandamide (AEA), 2-arachidonoylglycerol (2-AG) and OEA and PEA, (iii) and enzymes involved in synthesis (diacylglycerol lipase [DAGL], N-acylphosphatidyl-ethanolamine phospholipase D [NAPE-PLD]) and degradation (fatty acid amide hydrolase [FAAH] and monoacylglycerol lipase [MGL])^9^ of the endogenous ligands. Components of the ECS and the endocannabinoidome are involved in diverse mechanisms in the GI tract, such as food intake and satiation^12^, epithelial barrier integrity^13^ and immune tolerance^14^.

Despite a wealth of preclinical data demonstrating that the ECS controls the well-being and normal functioning of the GI tract, little evidence exists whether this also holds true for the human GI tract. Since the use of medicinal *Cannabis* has been introduced in several countries^15^, knowledge on the human ECS is of great importance for a potential therapy with cannabinoids. Surveys^16–19^ and observational/prospective studies^20–23^ revealed that IBD patients often self-medicate with *Cannabis* to alleviate abdominal pain and diarrhea. So far, only one small prospective human trial has investigated the effect of tetrahydrocannabinol (THC) in CD patients, who showed some kind of benefit^22^. Another trial investigated the effect of cannabidiol (CBD), a *Cannabis* constituent with little or no affinity to CB receptors^24^ in CD patients, revealing no beneficial effect^25^ despite the fact that CBD improves inflammation in animal models of IBD^5^. A survey by Storr *et al*. showed that *Cannabis* use for more than six months was even a strong predictor for surgery in CD patients^18^. Therefore, in contrast to preclinical studies, the role of the ECS in humans is not so clear. Several studies describe deregulation of ECS receptors, enzymes and endocannabinoids in colonic biopsies and whole tissue from IBD patients^26–28^. A change in ECS components in human CRC tissue samples was reported, indicating overproduction of endocannabinoids^29,30^ as well as high^30^ or low^31^ presence of CB_1_. High presence of CB_1_ was indicative of a poorer prognosis in stage II microsatellite stable tumour patients^32^ and stage IV CRC patients^33^. Also CB_2_ was shown to be a marker for poor prognosis in CRC patients^34^ although studies in mice have suggested a protective role for CB_1_^31,35^ and CB_2_^8^. In contrast, Ligresti *et al*. did not observe differences in CB_1_ and CB_2_ expression between normal and CRC tissue^29^. These contradicting results warrant closer examination of the ECS and the endocannabinoidome in both humans and mice.

In a recent study we could demonstrate that GPR55 plays a pro-oncogenic role in experimental CRC and described a significant association between high GPR55 expression and shortened relapse-free survival of CRC patients^35^. In our present study we have chosen to investigate levels of endocannabinoids, endocannabinoid-like lipids and lysophosphatidylinositol (LPI), the endogenous ligand of GPR55, in plasma of IBD and CRC patients to see whether deregulation of these metabolites is also detectable in the blood. This is of importance as they may become useful as biomarkers of disease. In addition, we took biopsies from patients with UC and CD, and from control subjects to measure gene expression of various receptors and enzymes of the ECS and the endocannabinoidome using NanoString technology. As many commercial antibodies against GPR55 and CB_2_ receptors lack specificity in immunohistochemical stainings^36,37^, we chose to investigate their localization in colonic biopsies of UC and CD patients and in colon cancer tissue by a novel and specific *in situ* hybridisation (ISH) technique (RNAscope) that we have successfully used in murine models of intestinal inflammation^37^.

## Results

### Differences in plasma endocannabinoid and endocannabinoid-like lipid levels between IBD/CRC patients and control subjects

**Figure 1:**
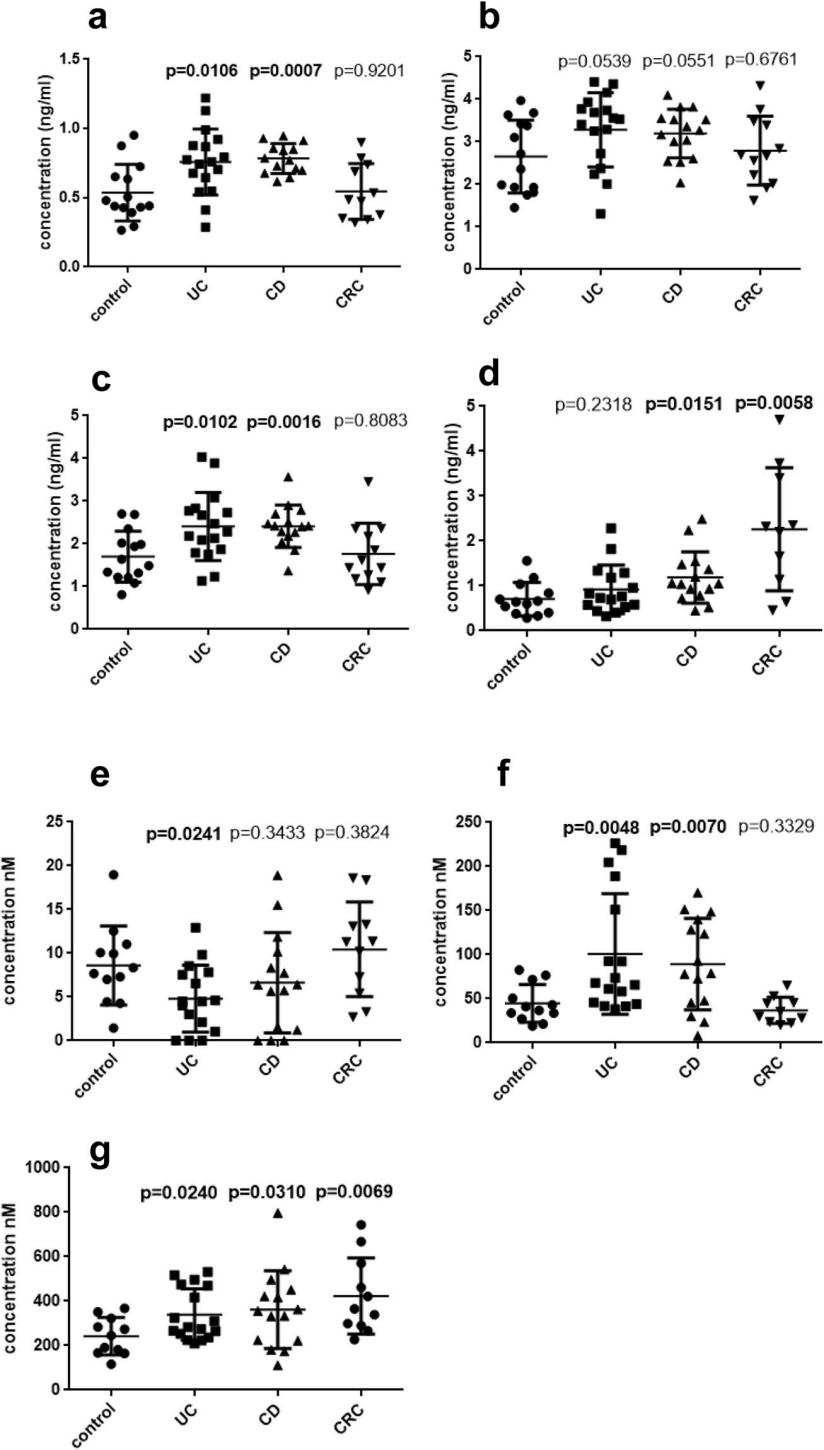
Mass spectrometry data of plasma endocannabinoid, endocannabinoid-like lipid and LPI levels. Levels (ng/ml) of anandamide (AEA) (**a**), palmitoyl- (PEA) (**b**) and oleoylethanolamide (OEA) (**c**), 2-arachidonoylglycerol (2-AG) (**d**), and of lysophosphatidylinositol (LPI) 16:0 (**e**), 18:0 (**f**) and 20:4 (**g**) are shown in plasma samples of patients with ulcerative colitis (UC), Crohn’s disease (CD), and colorectal cancer (CRC), each in comparison to control subjects. N = 12–17; Student’s t- test; p < 0.05 considered significant; data are means ± SD.

AEA, PEA and OEA were markedly increased in plasma of UC and CD (although PEA only slightly missed significance in UC (p = 0.0539) and CD (p = 0.0551) (Fig. 1a–c). The other main endocannabinoid, 2-AG, also showed an increase in IBD patients in comparison to control subjects, reaching significance (p = 0.0151) in CD, but not in UC (p = 0.2318; Fig. 1d). With regard to LPI (the endogenous GPR55 agonist), both LPI 18:0 and LPI with an arachidonic acid moiety (20:4) showed higher levels in IBD in comparison to control subjects (Fig. 1f–g). In contrast, levels of LPI 16:0 were lower in UC patients vs. controls (Fig. 1e). We observed an increase of 2-AG, but not of AEA, PEA and OEA, in CRC, while only LPI 20:4 and not LPI 18:0 and 16:0 was higher in CRC plasma in comparison to controls (Fig. 1e–g).

**Figure 2:**
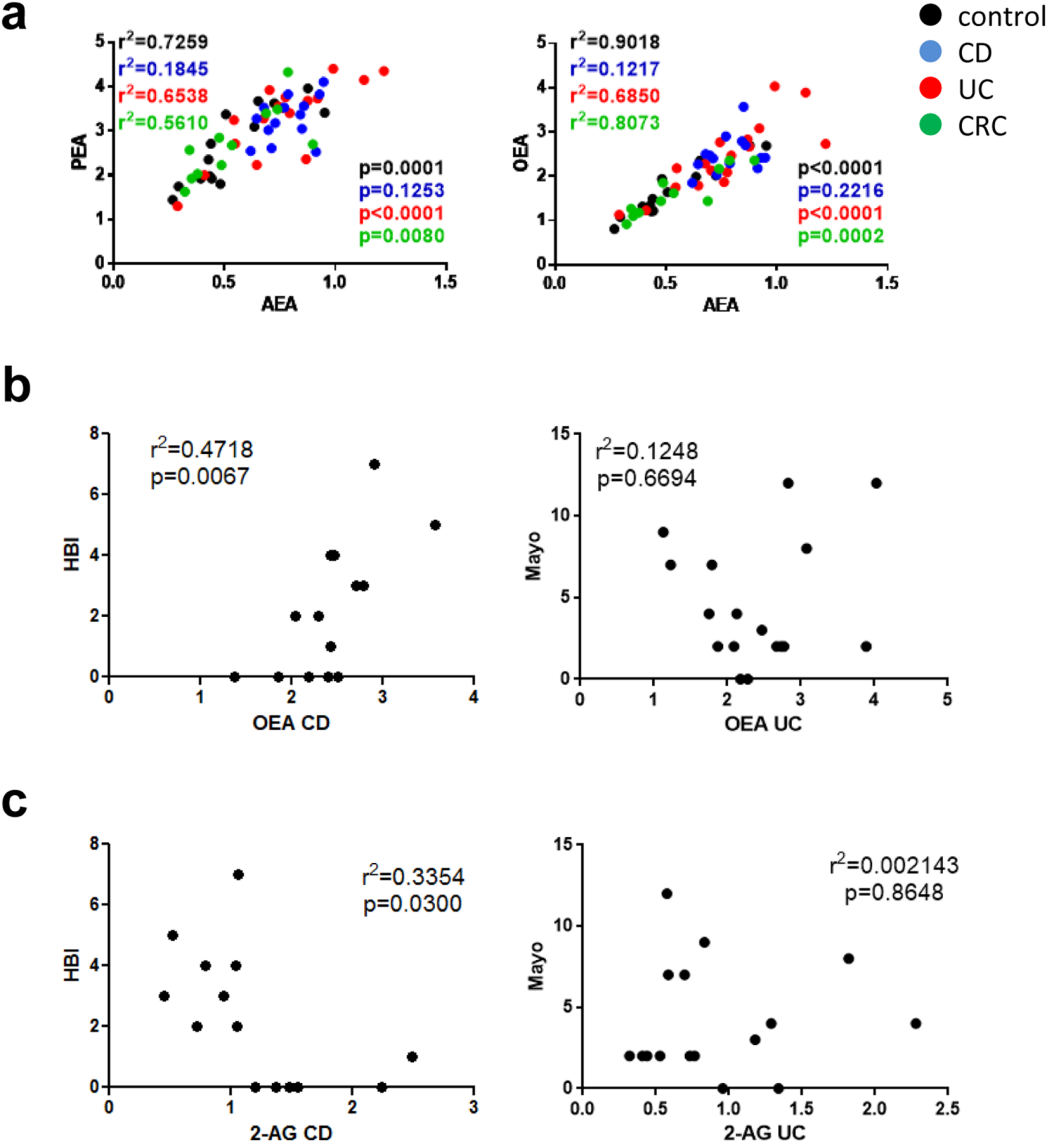
Correlations within endocannabinoid and endocannabinoid-like lipid levels and with parameters of disease severity. (**a**) Anandamide (AEA) levels (ng/ml) highly correlate with levels of palmitoylethanolamide (PEA) and oleoylethanolamide (OEA) (ng/ml) in plasma samples of controls and in patients with ulcerative colitis (UC) and colorectal cancer (CRC). (**b**) OEA (ng/ml) highly correlates with severity of CD (*Harvey Bradshaw Index*, HBI) but not with severity of UC (*Mayo* score). (**c**) 2-arachidonoylglycerol (2-AG) correlates negatively with HBI in CD, but not with Mayo score in UC. N = 12–17; Pearson correlation; p < 0.05 considered significant.

Consistent with the fact that they share similar synthetic and degradative pathways^38,39^ plasma concentrations of AEA, PEA and OEA highly correlated in control and diseased individuals (except for CD patients) indicating concerted production and metabolism of these lipids (Fig. 2a). We also wanted to know whether some of the endocannabinoids would correlate with the severity of CD and UC and we found that only OEA levels correlated with the Harvey Bradshaw Index (HBI) index in CD patients but not with the total Mayo score (which describes severity of UC) (Fig. 2b). Levels of 2-AG negatively correlated with the HBI of CD patients, but not with the total Mayo score of UC patients (Fig. 2c). Correlations of endocannabinoids and endocannabinoid-like lipids with the other parameters mentioned in Tables 1 and 2 for UC and CD patients (endoscopic Mayo score, SES-CD score, white blood cell count, C-reactive protein, calprotectin) had p values > 0.05.

**Table 1:** Characteristics and medication of UC patients.

Age	Disease duration (years)	Montreal Classification	Disease activity	Total Mayo Score	Endoscopic Mayo Score	WBC (x10^3^/L)	CRP (mg/L)	Calprotectin (µg/g)	BMI (kg/m^2^)	Current IBD specific therapy
31	10	E2	Remission	2	1	7.31	0.7	701	24.8	mesalamine
45	6	E1	Mild	3	1	4.95	0.8	<20	na	mesalamine, AZA
39	15	E3	Remission	0	0	6.07	1.0	62	23.0	vedolizumab
41	5	E2	Mild	4	2	6.27	1.4	647	28.4	mesalamine, steroids
66	32	E3	Remission	0	0	8.12	1.5	<20	24.8	mesalamine
68	4	E2	Moderate	7	2	10.47	39.2	502	23.1	mesalamine, steroids, antibiotics
53	0.8	E3	Severe	12	3	9.28	14.4	63	18.4	mesalamine, steroids, tacrolimus
48	11	E3	Remission	2	2	6.65	1.5	781	30.3	adalimumab
57	14	E2	Remission	2	0	6.66	3.5	218	19.7	mesalamine
39	2	E3	Moderate	8	3	6.66	5.0	2162	24.7	mesalamine
29	13	E2	Remission	2	1	8.76	1.3	272	15.5	mesalamine, AZA, adalimumab
63	13	E2	Mild	4	1	4.61	4.4	126	26.2	mesalamine, infliximab
33	2	E3	Moderate	9	3	16.99	73	2436	30.4	mesalamine, golimumab, antibiotics, *E. coli* Nissle
41	20	E3	Remission	2	1	7.62	2.1	<20	22.1	AZA
45	14	E2	Remission	2	1	7.75	4.8	671	22.1	mesalamine
22	7	E3	Severe	12	3	7.22	119.1	787	23.3	AZA
44	nd	E3	Moderate	7	2	7.72	2.9	na	22.3	none

*AZA*, azathioprine; *CRP*, C-reactive protein; *WBC*, white blood cell count; *BMI*, body mass index; *nd*, newly diagnosed; *na*, not applied.

**Table 2:** Characteristics and medication of CD patients.

Age	Disease duration (years)	Montreal Classification	HBI	SES-CD Score	CD endoscopic activity	WBC (x10^3^/L)	CRP (mg/L)	Calprotectin (µg/g)	BMI (kg/m^2^)	Current IBD specific therapy
33	10	A2L3B2	0	17	severe	9.3	9.7	1416	21.7	vedolizumab
50	5	A2L2B3p	3	0	inactive	7.87	6.6	<20	27.9	none
42	nd	A3L1B1	0	7	mild	10.06	7.2	872	25.2	mesalamine
33	10	A2L2B2	1	12	moderate	8.59	4.1	826	29.4	none
22	0.5	A2L3B2	4	24	severe	9.66	55.8	980	21.5	steroids, antibiotics
25	7	A2L4 + B1	2	0	inactive	9.98	<0.6	24	22.2	infliximab
44	6	A2L3B3	3	5	mild	10.26	9.9	284	18.9	adalimumab
35	16	A2L3B2	2	7	mild	5.33	3.5	155	27.4	adalimumab
46	3	A1L4 + B3p	0	3	inactive	6.09	2.1	938	20.9	infliximab
40	15	A2L1B2	4	3	inactive	3.56	0.7	60	24.2	antibiotics
36	11	A2L2B1	5	9	mild	8.85	3.6	2345	19.4	AZA
49	20	A2L1B2	0	6	mild	2.69	<0.6	240	19.8	AZA, adalimumab
43	0.7	A2L3B1	7	26	severe	14.45	49.5	<20	27.5	AZA, steroids, mesalamine
52	6	A2L3B3p	0	0	inactive	5.49	1.9	<20	20.1	cholestyramine
54	28	A2L2B1	7	10	mild	5.74	2.8	196	2713	mesalamine, adalimumab, AZA

*AZA*, azathioprine; *CRP*, C-reactive protein; *HBI*, Harvey-Bradshaw Index; *BMI*, body mass index; *nd*, newly diagnosed; *na*, not applied; *SES-CD*, Simple Endoscopic Score for Crohn’s Disease; *WBC*, white blood cell count.

### Differential expression of enzyme and receptor genes in mucosal biopsies of UC and CD patients

We further investigated mRNA of genes from endocannabinoid and endocannabinoid-like lipid synthesizing (*NAPE-PLD*, *DAGL*alpha and *DAGL*beta) and degrading enzymes (*FAAH*, *MGLL*, *NAAA*, *ABHD6*), as well as from CB (*CNR1*, *CNR2*) and non-CB receptors (*GPR18*, *GPR119*, *GPR55*, *TRPV1*, *PPAR*alpha, -gamma, and -delta).

**Figure 3:**
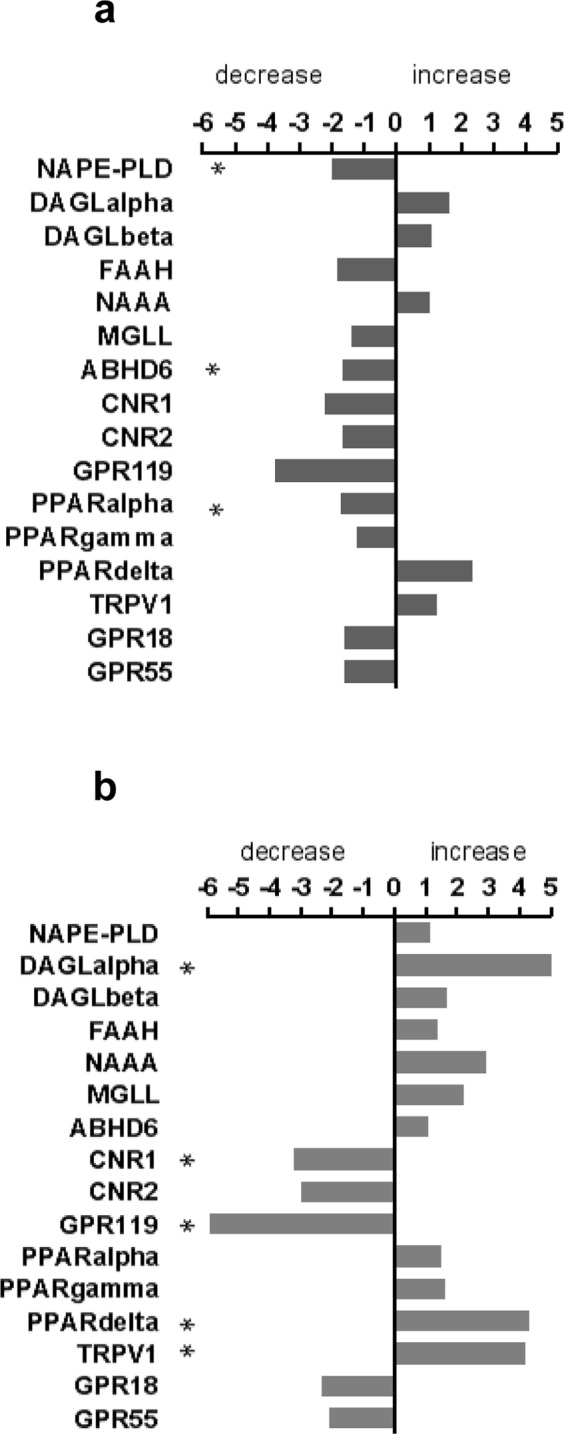
NanoString analysis of enzyme and receptor transcripts in intestinal mucosal biopsies. Graphs showing fold changes (decrease/increase) of mRNA of enzymes and receptors of the ECS and the endocannabinoidome in intestinal mucosal biopsies of patients with ulcerative colitis (UC) (**a**) and Crohn’s disease (CD) (**b**) in comparison to biopsies from control subjects. n = 9–11; One-Way ANOVA was performed between sample groups. Genes with p values < 0.05 and fold changes of at least 1.5 were considered to be significantly regulated (marked by *asterisks* *). See p values of each target in Supplementary Table S1.

Intestinal mucosal biopsies from UC and CD patients clearly showed differential patterns of gene expression. In general, the fold increases/decreases were more pronounced in CD than UC patients. Most of the investigated transcripts showed tendencies of decrease in UC patients (Fig. 3a) in comparison to the ones from controls, whereas in CD patients (Fig. 3b), it was the opposite for all endocannabinoid-synthesizing and -degrading enzymes investigated. Among the synthesizing enzymes, mRNA levels of NAPE-PLD were decreased in UC, whereas in CD, we observed an increase of DAGLalpha. In contrast to the enzymes, expression of receptors like *CNR1* (which encodes CB_1_) and *GPR119* were decreased in CD in comparison to controls whereas expression of *CNR2* (which encodes CB_2_), *GPR18*, and *GPR55* were less affected. Notably, *PPAR*delta increased in CD. As an AEA responsive receptor, *TRPV1* expression was higher in people with CD than in controls (Fig. 3b). For a complete list of fold changes and p values see Table S1 in the supplementary information.

### Localization of GPR55 and CB2 in IBD and CRC patients

**Figure 4:**
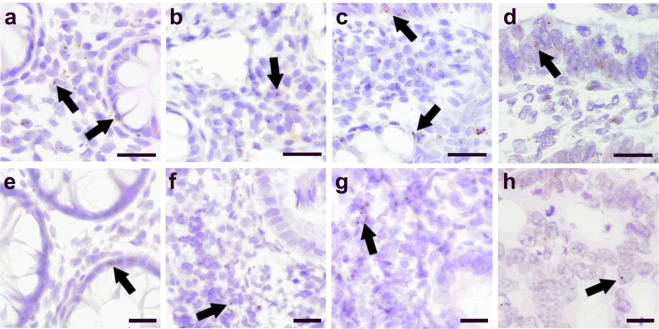
Localization of receptors of GPR55 and CB_2_ in IBD and CRC patients. ISH staining of GPR55 mRNA (**a**–**d**) and CB_2_ mRNA (**e**–**h**) in colonic mucosal biopsies of control subjects (**a**,**e**), patients with Crohn’s disease (CD) (**b**,**f**), ulcerative colitis (UC) (**c**,**g**), and colorectal cancer (CRC) (**d**,**h**). Images are representative for three individuals/cohort. Arrows point at representative DAB staining of GPR55 and CB_2_ mRNA. Calibration bars: 50 µm (**a**–**d**); 20 µm (**e**–**h**).

To demonstrate presence and distribution of a non-cannabinoid (but cannabinoid-responsive) G protein-coupled receptor and a cannabinoid receptor, we performed GPR55 and CB_2_ mRNA staining by ISH in sections of colonic mucosal biopsies, as depicted in Fig. 4. In tissue of control subjects, low levels of GPR55 mRNA were detected in few epithelial cells and in cells of the lamina propria, predominantly lymphocytes (Fig. 4a). Biopsies of CD patients showed very low mRNA staining in the lamina propria (Fig. 4b). In UC patients, GPR55 mRNA was generally low, but locally higher in epithelial cells (Fig. 4c, arrows). In CRC tissue, low GPR55 gene expression was detected in epithelial tumour and few other, possibly inflammatory, cells (Fig. 4d).

Low CB_2_ receptor mRNA levels were found in epithelial cells of control subjects (Fig. 4e) and in the lamina propria of CD patients (Fig. 4f) as well as UC patients (Fig. 4g). Low CB_2_ receptor mRNA was also detected in some tumour cells of tumour sections from CRC patients (Fig. 4h).

## Discussion

Although a considerable amount of knowledge has been gained from experimental models of intestinal inflammation and CRC about the protective effects of cannabinoids, there is still a wide gap between preclinical evidence and the role of the ECS in human GI diseases. So far, little evidence exists whether cannabinoids are beneficial in human IBD and CRC, as large clinical trials are still missing. Our study was, therefore, designed to measure changes within the members of the ECS and members now regarded as part of the endocannabinoidome (e.g. GPR55, GPR119, PPARs, PEA, OEA, LPI etc.)^11^ in human IBD and CRC to evaluate potential targets for pharmacological therapy. We also chose to measure endocannabinoids and several endocannabinoid-like lipids in plasma, a more convenient procedure to perform when determining biomarkers.

Preclinical studies with either MGL^40^ or FAAH inhibitors^41^, CB_1_ and CB_2_ receptor agonists^6,42–44^, GPR55 antagonists^45^, *Cannabis* extract and phytocannabinoids, such as cannabidiol^46–48^, β-caryophyllene^49,50^ and cannabigerol^51^, have all shown protection against experimental GI inflammation. These studies interestingly suggest that phytocannabinoids rather act via components of the endocannabinoidome than via the ECS. Additionally, treatment with PEA^52^ and AEA^53^ and with MGL inhibitors^40^ (to increase endogenous 2-AG) have demonstrated that GI inflammation can be reduced by exogenous as well as endogenous cannabinoids. Studies of experimental CRC also indicate anticarcinogenic effects of MGL inhibitors^54^, phytocannabinoids^7,8,55^ and the CB_1_ antagonist rimonabant^56^.

The theory states that the ECS is upregulated in inflammatory conditions to reinstate homeostasis^57^. According to our findings, this may be particularly true for AEA, PEA, and OEA, which were increased in IBD, but not in CRC. In line with our results, an increase of AEA (but not of 2-AG) and PEA was already described in colonic mucosal biopsies of UC patients in comparison to controls^58,59^. We also observed an increase of 2-AG in CD and, consistent with reports by Sailler *et al*.^60^, in CRC. Ligresti *et al*. also measured increased levels of AEA in colorectal tissue of CRC patients^29^. OEA has not yet been investigated in models of intestinal inflammation but seems to be part of a concerted upregulation of endocannabinoids and endocannabinoid-like lipids. That OEA shares pathways of synthesis and degradation with PEA and AEA^38,39^, is indicated by the close correlation of AEA, PEA and OEA levels (Fig. 2a). The increase in OEA is of considerable interest as it correlates with the HBI in CD patients. This endocannabinoid-like lipid is a most potent GPR119 agonist^61^. GPR119 has been linked with satiety and the brain-gut axis^62^, rather than with inflammation, but an article recently indicated a role in the prevention of steatohepatitis^63^. GPR119 may also mediate release of GLP-1^64^, a peptide that was shown to increase in DSS colitis^65^ and to promote anti-inflammatory mechanisms^66^. In our study, GPR119 was found downregulated in IBD patients, which may have been caused by the increased presence of OEA.

Next to endocannabinoids and endocannabinoid-like lipids, we were also interested in LPI, the endogenous ligand for GPR55, in order to understand how this ligand is regulated in IBD and CRC. Elevated levels of LPI have been shown in ovarian cancer^67^ and in a small cohort of colon cancer patients^68^. Stearoyl-LPI (18:0) and arachidonoyl-LPI (20:4) are the most abundant fatty acids in LPI, but LPI 20:4 was the most potent among them to produce responses in GPR55-HEK293 cells^69^. In mouse models of intestinal inflammation and CRC, GPR55 was demonstrated to act as a pro-inflammatory and pro-carcinogenic receptor^35,45^. The strong increase of LPI 18:4 and 20:4 in IBD and CRC may indicate an active LPI-GPR55 axis in these diseases. This concept is strongly supported by a new study showing increased LPI levels in mice with chronic and acute intestinal inflammation^70^. GPR55 transcripts, however, were not upregulated in the IBD cohorts probably indicating, similar to GPR119, receptor downregulation by increased presence of the ligand.

Evaluation of transcripts by NanoString analysis revealed differential regulation of endocannabinoid-synthesizing/-degrading enzymes in IBD patients. Most of all, expression levels of NAPE-PLD and DAGLalpha differed between UC and CD. The 2-AG producing enzyme DAGLalpha was increased in CD patients, which is in line with the high 2-AG levels measured in plasma. The purpose of high 2-AG availability may be to ameliorate inflammation, as suggested by Alhouayek *et al*.^40^ and our own findings (2-AG levels are lower in cases of a high HBI; Fig. 2b). In contrast, UC patients showed decreased NAPE-PLD expression but high levels of AEA, PEA and OEA. Although our results on NAPE-PLD expression are supported by immunohistochemical findings by Marquez *et al*.^27^, it is not quite clear why endocannabinoids and endocannabinoid-like lipids were increased while their synthesizing enzyme was downregulated. An allosteric feedback inhibition of NAPE-PLD by AEA (or PEA and OEA) or a reduced degradation of the endocannabinoids/-like lipids would be likely explanations although it is possible that N-acylethanolamine-producing enzymes other than NAPE-PLD were responsible for the increase of AEA, PEA and OEA^71^. ABHD6, a serine hydrolase known to regulate 2-AG efficacy at CB_2_ receptors^72^ was decreased in UC, but its role in the GI tract has not yet been elucidated. Recent reports state that ABHD6 may control 2-AG levels in macrophages and that inhibition of ABHD6 increase LPI 20:4 levels in LPS-activated macrophages^70,73^.

Most of the CB and non CB receptor transcripts, in particular, those of CB_1_ and GPR119, were downregulated in IBD (not significantly in UC). Although we should keep in mind that many of the CB and non CB receptors are expressed in lamina propria cells, which are sensitive to steroids and immunosuppressants, CB_1_ and GPR119 are mainly localized to epithelial, enteric neuronal and enteroendocrine cells of the gut^74,75^ suggesting that they should be little affected by immunosuppressants. Thus, one explanation for the decrease in CB_1_ gene expression could be that CB_1_ was downregulated as a response to high levels of its ligand AEA which could have led to desensitization, a common feature in GPCRs with high ligand exposure^76^.

By using immunohistochemistry and immunoassays, several studies performed in mucosal tissue of IBD patients all described upregulation of CB_1_^26,28^ and, therefore, they seem to be in contrast to our results. However, one of the reasons why we opted for evaluating transcripts instead of protein is the fact that many commercially available CB_1_^77^ and CB_2_^36^ antibodies are not specific. In addition, as CB_1_^78^ and CB_2_^79^ receptors can be internalized and recycled, receptor protein regulation may very well differ from regulation of transcripts. Additionally, ISH stainings did not indicate upregulation of CB2 mRNA (Fig. 4f).

mRNA of PPAR receptors, in particular of PPARalpha and PPARgamma, were also measured as they have been frequently implicated in anti-inflammatory actions of cannabinoids in intestinal epithelial cells^80^. Notably, they were regulated differentially in UC and CD.

Since we chose to study levels of endocannabinoids/-like lipids in plasma, the study has certain limitations and caveats. Plasma levels of endocannabinoids and endocannabinoid-like lipids can be associated with body mass index (BMI) and HDL-cholesterol levels^81^, however, this has been only shown for 2-AG and not for AEA, PEA and OEA in obese people with high, but not low intra-abdominal adiposity^81^. Also LPI levels were shown to be increased in obese people^82^. According to that study, obese people also have increased levels of LPI 16:0 which we did not measure in our disease cohorts. The mean BMIs of our UC (23.7 ± 4.0 kg/m^2^) and CD (23.3 ± 3.6 kg/m^2^) cohorts do not lie in the obesity range, indicating that obesity can be excluded as a confounding factor. Another caveat is the age difference between CRC patients and control subjects. But similar to our results, previous studies also showed increased 2-AG levels in colonic mucosal biopsies^29^ and plasma^60^ from CRC patients indicating that the differences we have seen in our samples are likely unrelated to age differences.

In summary, our data have highlighted the role of the ‘endocannabinoidome’ and in particular of the ECS in human IBD and CRC showing a distinct, but not general regulation of their components in IBD and CRC. The increase of endocannabinoids and endocannabinoid-like lipids in IBD seems to be a concerted action, but only OEA and 2-AG correlated, although oppositely, with disease severity in CD patients. In addition, regulation of endocannabinoid-synthesizing/-degrading enzymes is different to CB receptor expression. The study also suggests that LPI may have a proinflammatory role in human IBD, supported by recent data of murine intestinal inflammation^70^. Although frequently suggested as a homeostatic mechanism to injury in the GI tract, we failed to see an upregulation of CB receptor mRNA in the colon of IBD patients by NanoString. As many herbal and synthetic cannabinoid (also non-psychotropic) products are available, larger clinical trials are worthwhile to test their efficacy in GI diseases

## Methods

### Patients

Adult patients with confirmed UC (n = 17; mean age ± SD: 45 ± 13.1; 8 males/9 females), CD (n = 15; mean age ± SD: 40.3 ± 9.5; 11 males/4 females), CRC (n = 12; mean age ± SD: 75.5 ± 10.3; 10 males/2 females) and control subjects (n = 19; mean age ± SD: 34.6 ± 15.1; 6 males/13 females) were included in the study. UC and CD patients were recruited from the IBD clinic of the Department of Internal Medicine at the Medical University of Graz; CRC patients were recruited as part of the OncoTrack project (http://www.oncotrack.eu/) by the General Hospital Graz West and the St. John of God Hospital Graz, control subjects were recruited from the endoscopy unit of the Department of Internal Medicine and the Division of Pharmacology, Medical University of Graz. For the measurement of endocannabinoids and LPI in plasma, blood was collected in Vacuette EDTA tubes (Greiner-Bio-One, Austria), centrifuged at 1500xg for 15 min, aliquoted and frozen at −80 °C until use.

Diagnosis of UC and CD was established by standard clinical, endoscopic and histologic criteria^83,84^. All UC and CD patients with active disease also underwent contemporaneous colonoscopy to assess endoscopic disease activity. For UC patients, disease activity were assessed using the total Mayo score and endoscopic activity by the endoscopy Mayo subscore^85^. For CD patients, activity was assessed clinically by the Harvey Bradshaw Index (HBI) and endoscopically by the Simple Endoscopic Score for Crohn’s Disease (SES-CD)^86–88^. Endoscopic biopsies were collected from inflamed intestinal mucosal area from UC and CD patients. Colonoscopy was performed as part of the clinical workup because of active disease. Normal colonic biopsies were obtained from a control group with a normal colonoscopy recruited from patients undergoing colonoscopy as part of the colon cancer screening program and from patients with diagnostic workup of occult or overt gastrointestinal bleeding. All subjects were recruited from the endoscopy unit of the Department of Internal Medicine, Medical University of Graz. Those with significant comorbidities, intercurrent illness such as infections, and pregnant women were excluded from the study. Collected biopsies were immediately frozen or fixed in 10% phosphate buffered formalin for histochemical analysis. All subjects suffering from UC and CD were on some sort of medication. Characteristics and medication of CD and UC patients are shown in Tables 1 and 2. Characteristics of CRC patients are shown in Table 3. UICC classifications are according to Sobin^89^.

**Table 3:** Characteristics of CRC patients.

Age	male/female	Stage*	TNM*	Tumor origin
86	f	IIA	T3 N0 M0	Colon
74	m	IIIB	T3 N1a M0	Colon
79	m	IIIC	T3 N2b M0	Colon
75	m	IIA	T3 N0 M0	Colon
66	m	I	T2 N0 M0	Colon
91	m	I	T1 N0 M0	Colon
79	f	I	T2 N0 M0	Rectum
55	m	I	T1 N0 M0	Rectum
77	m	I	T1 N0 M0	Rectum
66	m	I	T2 N0 M0	Rectum
88	m	IV	T4a N2a M1a	Colon
70	m	I	T2 N0 M0	Colon

^*^According to UICC classifications.

#### Ethical approval and informed consent

Ethical approval was granted by the ethics committee of the Medical University of Graz and confirmed by the ethics committee of the St John of God Hospital Graz (protocol numbers: 24–281 ex 11/12; 23-015 ex 10/11; 17–291 ex 05/06 and 23-002 ex 10/11). Procedures were carried out in accordance with international guidelines. All participants provided written, informed consent. All samples and medical data used in this study were irreversibly anonymized.

#### Determination of endocannabinoids, endocannabinoid-like lipids and LPI in plasma by LC-MS measurement

The determination of the endocannabinoids AEA and 2-AG, the structurally related compound 1-arachidonoyl glycerol (1-AG), and of the endocannabinoid-like lipids OEA and PEA was performed by liquid-liquid-extraction in combination with ultra-high-performance liquid chromatography-tandem mass spectrometry (UHPLC-MS/MS). Reference substances and internal standards were provided by Cayman Chemical (Ann Arbor, MI, USA). For all analytes isotopically labeled analogues were used as internal standards. Working solutions were prepared in acetonitrile as mixture of all analytes and internal standards, respectively.

The LC-MS/MS system consisted of a triple quadrupole mass spectrometer QTRAP 6500+ (Sciex, Darmstadt, Germany) equipped with a Turbo Ion Spray source operated in positive electrospray ionization mode and an Agilent 1290 Infinity LC-system with binary HPLC pump, column oven and autosampler (Agilent, Waldbronn, Germany). The chromatographic separation was performed using an Acquity UPLC BEH C18 2.1 × 100 mm column (particle size of 1.7 µm, Waters, Eschborn, Germany) in combination with an UHPLC Fully Porous C18 column guard (Phenomenex, Aschaffenburg, Germany). A gradient elution using water with 0.0025% formic acid (solvent A) and acetonitrile with 0.0025% formic acid (solvent B) was applied.

Plasma samples were thawed in a refrigerator and processed on ice all the time. The sample (100 µL) was mixed with 20 µL acetonitrile and 20 µL IS working solution. The mixture was vortexed and centrifuged. Afterwards the mixture was extracted once with 200 µL ethyl acetate/hexane (9/1, v/v). The upper phase was removed after vortexing and centrifuging and evaporated at 45 °C under a gentle stream of nitrogen. The sample was reconstituted in 50 µL acetonitrile. Phosphate-buffered-saline (PBS) was used as surrogate matrix for the preparation of calibration standards and quality control samples. A volume of 100 µl PBS was spiked with 20 µL of a working solution and processed as stated before starting with the addition of 20 µL IS working solution.

Data acquisition was done using Analyst Software 1.6.3 and quantification was performed with MultiQuant Software 3.0.2 (both Sciex, Darmstadt, Germany), employing the internal standard method (isotope dilution mass spectrometry). Calibration curves were calculated by linear regression with 1/x weighting.

Further information on the used gradient, mass spectrometric parameters, precursor to product ion transitions (m/z), internal standards as well as the lower and upper limit of quantification can be found in the Supplementary Table S2.

For LPI measurement, lipid was extracted according to Matyash *et al*.^90^. Liquid chromatography-mass spectrometry (LC-MS) measurements for LPI quantification and data processing were performed with slight modifications as previously described in Triebl *et al*.^91^ and Hartler *et al*.^92^. See Supplementary Information S3 for a detailed description.

### NanoString analysis for intestinal mucosal biopsies

#### Sample Collection and total RNA Isolation

Collected biopsy samples were immediately frozen and kept on −80 °C until use. Total RNA was isolated with the Qiagen miRNeasy Micro Kit (Qiagen, Hilden, Germany) according to manufacturer’s instructions, quantified on a NanoDrop 2000c Spectrophotometer and RNA integrity numbers (RIN) were calculated for all samples on an Agilent BioAnalyzer 2100 instrument.

#### NanoString nCounter Custome CodeSet hybridisation

Total RNA with RIN values between 2.2 and 5.5 were used for nCounter GX Custom CodeSet hybridisation according to manufacturer’s instructions (NanoString Technologies, Seattle, WA USA). Briefly, 400 ng to 800 ng of total RNA per sample were used with an nCounter GX Custom CodeSet to detect 16 target genes and 2 reference genes (hypoxanthine phosphoribosyltransferase [*HPRT*] and hydroxymethylbilane synthase [*HMBS*]) hybridized for 19 hours over night and processed on an nCounter MAX prep station (NanoString Technologies, Seattle, WA USA). Cartridges were scanned with an nCounter® Digital Analyzer (NanoString Technologies, Seattle, WA USA). Samples were processed in the Core Facility Molecular Biology at the Centre of Medical Research at the Medical University of Graz, Austria.

### RNAscope *in situ* hybridisation (ISH)

Tissue was fixed and dehydrated, embedded in paraffin, cut in 5 μm sections, and finally dried and stored before submitting it to RNAscope ISH.

RNAscope is an advanced ISH method where two adjacent probes (so-called ZZ probes) are needed to bind to the target sequence to develop a signal. This method provides the possibility of detecting a low number of mRNAs in peripheral tissue due to a decreased background noise. Twenty ZZ probes for human CB_2_ (targeting bases 141–1193 of NM_001841.2) and GPR55 (targeting bases 2–1057 of NM_005683.3) (purchased from Advanced Cell Diagnostics [ACD] Newark, USA) were used to detect the corresponding mRNAs in intestinal mucosal biopsies of controls, IBD and CRC patients. The ISH was performed according to the manufacturer’s protocol by using RNAscope 2.5 HD brown kit (ACD).

In brief, tissue sections were baked at 60 °C for 1 h, de-waxed, rehydrated and treated with H_2_O_2_ for 10 min. Target retrieval was performed by cooking of tissue in retrieval buffer using the Brown FS3000 food steamer for 15 min, each step followed by washes in H_2_O dest. The next day, sections were digested with Protease Plus at 40 °C for 20 min, washed, followed by incubation with the corresponding probes at 40 °C for 2 h. The procedure was continued according to the manufacturer’s protocol. CB_2_ and GPR55 mRNAs were stained using 3,3’-diaminobenzidine (DAB; provided by ACD). Sections were counterstained with 1:5 dilutions of Gill’s II hematoxylin, washed, dried and mounted with Vectamount mounting medium (Vector Laboratories). Sections from patients and control subjects were put on one slide for comparison. Brightfield images were taken using an Olympus BX41 microscope and an Olympus UC 90 digital camera connected with Olympus CellSense standard 1.17 imaging software (Olympus, Vienna, Austria). Contrast, brightness and color balance of images were adjusted using Corel Photo Paint.

### Statistics

#### Mass spectrometry data analysis

Data are shown as mean +/− standard deviation (SD). Statistical analysis was performed using GraphPad Prism 7.05 (GraphPad Software, La Jolla, CA, USA). Cohorts of control subjects and patients were compared by unpaired two-tailed Student’s t-test. For data with varying SDs, two-tailed t-test with Welsh correction was applied. Normal distribution of the data was tested using d’Agostino and Pearson omnibus normality test. Single outliers were detected using Grubb’s test. For LPI 20:4 measurement in control subjects, two outliers were detected using the ROUT method (Q = 5%) and excluded. For 2-AG measurement, 3 samples were below/above the detection limit and were, therefore, omitted. P values < 0.05 were considered significant.

#### Correlations

Data were subjected to normality test (d’Agostino & Pearson omnibus). In case of normal distribution, Pearson correlation was performed (deployed for all correlations shown). For groups without normal distribution, nonparametric correlation (Spearman) was applied.

#### NanoString data analysis

RCC raw data files were imported to nSolver 4.0 Software (NanoString Technologies, Seattle, WA USA) and raw data pre-processing and normalization was performed according to standard procedures (positive control and codeset normalisation using reference genes). Log2 normalized data were imported to Partek Genomic Suite Software v6.6 (Partek Inc, St Louis, MO) for statistical analysis. One-Way ANOVA was performed between sample groups. Genes with p values < 0.05 and fold changes of at least 1.5 were considered to be significantly regulated.

## Supplementary information

Supplementary information

## References

[1] Cosnes J, Gower-Rousseau C, Seksik P, Cortot A (2011). Epidemiology and natural history of inflammatory bowel diseases. Gastroenterology.

[2] Frolkis AD (2013). Risk of surgery for inflammatory bowel diseases has decreased over time: a systematic review and meta-analysis of population-based studies. Gastroenterology.

[3] Safroneeva E (2015). Prevalence and Risk Factors for Therapy Escalation in Ulcerative Colitis in the Swiss IBD Cohort Study. Inflamm. Bowel Dis..

[4] Terzic J, Grivennikov S, Karin E, Karin M (2010). Inflammation and colon cancer. Gastroenterology.

[5] Hasenoehrl C, Taschler U, Storr M, Schicho R (2016). The gastrointestinal tract - a central organ of cannabinoid signaling in health and disease. Neurogastroenterol. Motil..

[6] Massa F (2004). The endogenous cannabinoid system protects against colonic inflammation. J. Clin. Invest..

[7] Aviello G (2012). Chemopreventive effect of the non-psychotropic phytocannabinoid cannabidiol on experimental colon cancer. J. Mol. Med. (Berl).

[8] Romano B (2014). Inhibition of colon carcinogenesis by a standardized Cannabis sativa extract with high content of cannabidiol. Phytomedicine.

[9] Alhouayek M, Muccioli GG (2012). The endocannabinoid system in inflammatory bowel diseases: from pathophysiology to therapeutic opportunity. Trends Mol. Med..

[10] Leinwand KL, Gerich ME, Hoffenberg EJ, Collins CB (2017). Manipulation of the Endocannabinoid System in Colitis: A Comprehensive Review. Inflamm. Bowel Dis..

[11] Di Marzo V, Piscitelli F (2015). The Endocannabinoid System and its Modulation by Phytocannabinoids. Neurotherapeutics.

[12] DiPatrizio NV (2016). Endocannabinoids in the Gut. Cannabis Cannabinoid Res..

[13] Karwad MA (2017). The role of CB1 in intestinal permeability and inflammation. FASEB J..

[14] Acharya N (2017). Endocannabinoid system acts as a regulator of immune homeostasis in the gut. Proc. Natl. Acad. Sci. USA.

[15] Abuhasira R, Shbiro L, Landschaft Y (2018). Medical use of cannabis and cannabinoids containing products - Regulations in Europe and North America. Eur. J. Intern. Med..

[16] Lal S (2011). Cannabis use amongst patients with inflammatory bowel disease. Eur. J. Gastroenterol. Hepatol..

[17] Ravikoff Allegretti J, Courtwright A, Lucci M, Korzenik JR, Levine J (2013). Marijuana use patterns among patients with inflammatory bowel disease. Inflamm. Bowel Dis..

[18] Storr M, Devlin S, Kaplan GG, Panaccione R, Andrews CN (2014). Cannabis use provides symptom relief in patients with inflammatory bowel disease but is associated with worse disease prognosis in patients with Crohn’s disease. Inflamm. Bowel Dis..

[19] Phatak UP, Rojas-Velasquez D, Porto A, Pashankar DS (2017). Prevalence and Patterns of Marijuana Use in Young Adults With Inflammatory Bowel Disease. J. Pediatr. Gastroenterol. Nutr..

[20] Lahat A, Lang A, Ben-Horin S (2012). Impact of cannabis treatment on the quality of life, weight and clinical disease activity in inflammatory bowel disease patients: a pilot prospective study. Digestion.

[21] Naftali T, Lev LB, Yablecovitch D, Half E, Konikoff FM (2011). Treatment of Crohn’s disease with cannabis: an observational study. Isr. Med. Assoc. J..

[22] Naftali T (2013). Cannabis induces a clinical response in patients with Crohn’s disease: a prospective placebo-controlled study. Clin. Gastroenterol. Hepatol..

[23] Weiss A, Friedenberg F (2015). Patterns of cannabis use in patients with Inflammatory Bowel Disease: A population based analysis. Drug Alcohol Depend..

[24] Ryberg E (2007). The orphan receptor GPR55 is a novel cannabinoid receptor. Br. J. Pharmacol..

[25] Naftali T (2017). Low-Dose Cannabidiol Is Safe but Not Effective in the Treatment for Crohn’s Disease, a Randomized Controlled Trial. Dig. Dis. Sci..

[26] Stintzing S (2011). Role of cannabinoid receptors and RAGE in inflammatory bowel disease. Histol. Histopathol..

[27] Marquez L (2009). Ulcerative colitis induces changes on the expression of the endocannabinoid system in the human colonic tissue. PLoS One.

[28] Di Sabatino A (2011). The endogenous cannabinoid system in the gut of patients with inflammatory bowel disease. Mucosal Immunol..

[29] Ligresti A (2003). Possible endocannabinoid control of colorectal cancer growth. Gastroenterology.

[30] Chen L (2015). Endocannabinoid and ceramide levels are altered in patients with colorectal cancer. Oncol. Rep..

[31] Wang D (2008). Loss of cannabinoid receptor 1 accelerates intestinal tumor growth. Cancer Res..

[32] Gustafsson SB, Lindgren T, Jonsson M, Jacobsson SO (2009). Cannabinoid receptor-independent cytotoxic effects of cannabinoids in human colorectal carcinoma cells: synergism with 5-fluorouracil. Cancer Chemother. Pharmacol..

[33] Jung CK (2013). Expression of the cannabinoid type I receptor and prognosis following surgery in colorectal cancer. Oncol. Lett..

[34] Martinez-Martinez E (2015). Cannabinoids receptor type 2, CB2, expression correlates with human colon cancer progression and predicts patient survival. Oncoscience.

[35] Hasenoehrl C (2018). G protein-coupled receptor GPR55 promotes colorectal cancer and has opposing effects to cannabinoid receptor 1. Int. J. Cancer.

[36] Marchalant Y, Brownjohn PW, Bonnet A, Kleffmann T, Ashton JC (2014). Validating Antibodies to the Cannabinoid CB2 Receptor: Antibody Sensitivity Is Not Evidence of Antibody Specificity. J. Histochem. Cytochem..

[37] Grill, M., Hasenoehrl, C., Kienzl, M., Kargl, J. & Schicho, R. Cellular localization and regulation of receptors and enzymes of the endocannabinoid system in intestinal and systemic inflammation. *Histochem*. *Cell Biol* (2018). 10.1007/s00418-018-1719-0 PMC6328631 30196316

[38] Petrosino S, Iuvone T, Di Marzo V (2010). N-palmitoyl-ethanolamine: Biochemistry and new therapeutic opportunities. Biochimie.

[39] Borrelli F, Izzo AA (2009). Role of acylethanolamides in the gastrointestinal tract with special reference to food intake and energy balance. Best Pract. Res. Clin. Endocrinol. Metab..

[40] Alhouayek M, Lambert DM, Delzenne NM, Cani PD, Muccioli GG (2011). Increasing endogenous 2-arachidonoylglycerol levels counteracts colitis and related systemic inflammation. FASEB J..

[41] Salaga M (2014). Experimental colitis in mice is attenuated by changes in the levels of endocannabinoid metabolites induced by selective inhibition of fatty acid amide hydrolase (FAAH). J. Crohns Colitis.

[42] Kimball ES, Schneider CR, Wallace NH, Hornby PJ (2006). Agonists of cannabinoid receptor 1 and 2 inhibit experimental colitis induced by oil of mustard and by dextran sulfate sodium. Am. J. Physiol. Gastrointest. Liver Physiol..

[43] Storr MA (2009). Activation of the cannabinoid 2 receptor (CB2) protects against experimental colitis. Inflamm. Bowel Dis..

[44] Feng YJ, Li YY, Lin XH, Li K, Cao MH (2016). Anti-inflammatory effect of cannabinoid agonist WIN55, 212 on mouse experimental colitis is related to inhibition of p38MAPK. World J. Gastroenterol..

[45] Stancic A (2015). The GPR55 antagonist CID16020046 protects against intestinal inflammation. Neurogastroenterol. Motil..

[46] Pagano E (2016). An Orally Active Cannabis Extract with High Content in Cannabidiol attenuates Chemically-induced Intestinal Inflammation and Hypermotility in the Mouse. Front. Pharmacol..

[47] Borrelli F (2009). Cannabidiol, a safe and non-psychotropic ingredient of the marijuana plant Cannabis sativa, is protective in a murine model of colitis. J. Mol. Med. (Berl).

[48] Schicho R, Storr M (2012). Topical and systemic cannabidiol improves trinitrobenzene sulfonic acid colitis in mice. Pharmacology.

[49] Gertsch J (2008). Beta-caryophyllene is a dietary cannabinoid. Proc. Natl. Acad. Sci. USA.

[50] Bento AF (2011). beta-Caryophyllene inhibits dextran sulfate sodium-induced colitis in mice through CB2 receptor activation and PPARgamma pathway. Am. J. Pathol..

[51] Borrelli F (2013). Beneficial effect of the non-psychotropic plant cannabinoid cannabigerol on experimental inflammatory bowel disease. Biochem. Pharmacol..

[52] Esposito G (2014). Palmitoylethanolamide improves colon inflammation through an enteric glia/toll like receptor 4-dependent PPAR-alpha activation. Gut.

[53] Engel MA (2008). Ulcerative colitis in AKR mice is attenuated by intraperitoneally administered anandamide. J. Physiol. Pharmacol..

[54] Pagano E (2017). Pharmacological inhibition of MAGL attenuates experimental colon carcinogenesis. Pharmacol. Res..

[55] Borrelli F (2014). Colon carcinogenesis is inhibited by the TRPM8 antagonist cannabigerol, a Cannabis-derived non-psychotropic cannabinoid. Carcinogenesis.

[56] Santoro A (2009). Rimonabant inhibits human colon cancer cell growth and reduces the formation of precancerous lesions in the mouse colon. Int. J. Cancer.

[57] Di Marzo V, Izzo AA (2006). Endocannabinoid overactivity and intestinal inflammation. Gut.

[58] D’Argenio G (2006). Up-regulation of anandamide levels as an endogenous mechanism and a pharmacological strategy to limit colon inflammation. FASEB J..

[59] Darmani NA (2005). Involvement of the cannabimimetic compound, N-palmitoyl-ethanolamine, in inflammatory and neuropathic conditions: review of the available pre-clinical data, and first human studies. Neuropharmacology.

[60] Sailler S (2014). Regulation of circulating endocannabinoids associated with cancer and metastases in mice and humans. Oncoscience.

[61] Syed SK (2012). Regulation of GPR119 receptor activity with endocannabinoid-like lipids. Am. J. Physiol. Endocrinol. Metab..

[62] Hansen, H. S. & Vana, V. Non-endocannabinoid N-acylethanolamines and 2-monoacylglycerols in the intestine. *Br*. *J*. *Pharmacol* (2018). 10.1111/bph.14175 PMC6487557 29473944

[63] Bahirat, U. A., Talwar, R., Shenoy, R. R., Nemmani, K. V. S. & Goel, R. N. Combination of APD668, a G protein-coupled receptor 119 agonist with linagliptin, a DPPIV inhibitor, prevents progression of steatohepatitis in a murine model of non-alcoholic steatohepatitis with diabetes. *Med*. *Mol*. *Morphol* (2018). 10.1007/s00795-018-0200-4 29959534

[64] Lan H (2012). Agonists at GPR119 mediate secretion of GLP-1 from mouse enteroendocrine cells through glucose-independent pathways. Br. J. Pharmacol..

[65] Lebrun LJ (2017). Enteroendocrine L Cells Sense LPS after Gut Barrier Injury to Enhance GLP-1 Secretion. Cell. Rep..

[66] Lee YS, Jun HS (2016). Anti-Inflammatory Effects of GLP-1-Based Therapies beyond Glucose Control. Mediators Inflamm..

[67] Shen Z (2001). Fatty acid composition of lysophosphatidic acid and lysophosphatidylinositol in plasma from patients with ovarian cancer and other gynecological diseases. Gynecol. Oncol..

[68] Kargl J (2016). GPR55 promotes migration and adhesion of colon cancer cells indicating a role in metastasis. Br. J. Pharmacol..

[69] Oka S (2009). 2-Arachidonoyl-sn-glycero-3-phosphoinositol: a possible natural ligand for GPR55. J. Biochem..

[70] Masquelier J (2018). Lysophosphatidylinositols in inflammation and macrophage activation: Altered levels and anti-inflammatory effects. Biochim. Biophys. Acta Mol. Cell. Biol. Lipids.

[71] Leung D, Saghatelian A, Simon GM, Cravatt BF (2006). Inactivation of N-acyl phosphatidylethanolamine phospholipase D reveals multiple mechanisms for the biosynthesis of endocannabinoids. Biochemistry.

[72] Marrs WR (2010). The serine hydrolase ABHD6 controls the accumulation and efficacy of 2-AG at cannabinoid receptors. Nat. Neurosci..

[73] Alhouayek M, Masquelier J, Cani PD, Lambert DM, Muccioli GG (2013). Implication of the anti-inflammatory bioactive lipid prostaglandin D2-glycerol ester in the control of macrophage activation and inflammation by ABHD6. Proc. Natl. Acad. Sci. USA.

[74] Wright K (2005). Differential expression of cannabinoid receptors in the human colon: cannabinoids promote epithelial wound healing. Gastroenterology.

[75] Hansen HS, Rosenkilde MM, Holst JJ, Schwartz TW (2012). GPR119 as a fat sensor. Trends Pharmacol. Sci..

[76] Rajagopal S, Shenoy SK (2018). GPCR desensitization: Acute and prolonged phases. Cell. Signal..

[77] Grimsey NL (2008). Specific detection of CB1 receptors; cannabinoid CB1 receptor antibodies are not all created equal!. J. Neurosci. Methods.

[78] Hsieh C, Brown S, Derleth C, Mackie K (1999). Internalization and recycling of the CB1 cannabinoid receptor. J. Neurochem..

[79] Grimsey NL, Goodfellow CE, Dragunow M, Glass M (2011). Cannabinoid receptor 2 undergoes Rab5-mediated internalization and recycles via a Rab11-dependent pathway. Biochim. Biophys. Acta.

[80] O’Sullivan SE (2016). An update on PPAR activation by cannabinoids. Br. J. Pharmacol..

[81] Cote M (2007). Circulating endocannabinoid levels, abdominal adiposity and related cardiometabolic risk factors in obese men. Int. J. Obes. (Lond).

[82] Moreno-Navarrete JM (2012). The L-alpha-lysophosphatidylinositol/GPR55 system and its potential role in human obesity. Diabetes.

[83] Magro F (2017). Third European Evidence-based Consensus on Diagnosis and Management of Ulcerative Colitis. Part 1: Definitions, Diagnosis, Extra-intestinal Manifestations, Pregnancy, Cancer Surveillance, Surgery, and Ileo-anal Pouch Disorders. J. Crohns Colitis.

[84] Gomollon F (2017). 3rd European Evidence-based Consensus on the Diagnosis and Management of Crohn’s Disease 2016: Part 1: Diagnosis and Medical Management. J. Crohns Colitis.

[85] D’Haens G (2007). A review of activity indices and efficacy end points for clinical trials of medical therapy in adults with ulcerative colitis. Gastroenterology.

[86] Sandborn WJ (2002). A review of activity indices and efficacy endpoints for clinical trials of medical therapy in adults with Crohn’s disease. Gastroenterology.

[87] Daperno M (2004). Development and validation of a new, simplified endoscopic activity score for Crohn’s disease: the SES-CD. Gastrointest. Endosc..

[88] Schoepfer AM (2010). Fecal calprotectin correlates more closely with the Simple Endoscopic Score for Crohn’s disease (SES-CD) than CRP, blood leukocytes, and the CDAI. Am. J. Gastroenterol..

[89] Sobin, L., Gospodarowicz, M. & Wittekind, C. TNM Classification of Malignant Tumours, 7th Edition, **336** (2011).

[90] Matyash V, Liebisch G, Kurzchalia TV, Shevchenko A, Schwudke D (2008). Lipid extraction by methyl-tert-butyl ether for high-throughput lipidomics. J. Lipid Res..

[91] Triebl A, Trotzmuller M, Hartler J, Stojakovic T, Kofeler HC (2017). Lipidomics by ultrahigh performance liquid chromatography-high resolution mass spectrometry and its application to complex biological samples. J. Chromatogr. B. Analyt Technol. Biomed. Life. Sci..

[92] Hartler J (2017). Deciphering lipid structures based on platform-independent decision rules. Nat. Methods.

