# Supplementary material for: Members of the endocannabinoid system are distinctly regulated in inflammatory bowel disease and colorectal cancer
Source: Sci Rep. 2019 Feb 20;9:2358. doi: 10.1038/s41598-019-38865-4 (PMC6382821; doi:10.1038/s41598-019-38865-4)
Supplement: Supplementary file 1 — Supplementary information [file 41598_2019_38865_MOESM1_ESM.docx]

Members of the endocannabinoid system are distinctly regulated in inflammatory bowel disease and colorectal cancer

Magdalena Grill^1^, Christoph Högenauer^2,3^, Andreas Blesl^2^, Johannes Haybaeck^4,5,6^, Nicole Golob-Schwarzl^5^, Nerea Ferreirós^7^, Dominique Thomas^7^, Robert Gurke^7^, Martin Trötzmüller^8^, Harald C. Köfeler^8,9^, Birgit Gallé^10^, Rudolf Schicho^1,3,*^

^1^ Otto Loewi Research Center, Division of Pharmacology, Medical University of Graz, Graz, Austria.

^2^ Division of Gastroenterology and Hepatology, Department of Internal Medicine, Medical University

of Graz, Graz, Austria

^3^ BioTechMed, Graz, Austria

^4^ Department of Pathology, Otto von Guericke University, Magdeburg, Germany

^5^ Diagnostic and Research Institute of Pathology, Medical University of Graz, Graz, Austria

^6^ Department of Pathology, Medical University of Innsbruck, Innsbruck, Austria

^7^ Institute of Clinical Pharmacology, Goethe University, Frankfurt/Main, Germany

^8^ Core Facility for Mass Spectrometry, Center for Medical Research, Medical University of

Graz, Graz, Austria

^9^ Omics Center Graz, BioTechMed-Graz, 8010 Graz, Austria

^10^ Core Facility Molekularbiologie, Center for Medical Research, Medical University of Graz, Graz,

Austria

* Corresponding author:

**Rudolf Schicho, PhD**

Otto Loewi Research Center, Division of Pharmacology, Medical University of Graz, Universitätsplatz 4/I, 8010 Graz, Austria

phone: +43316-385-74114

fax: +43316-385-79613

email: rudolf.schicho@medunigraz.at

Supplementary Table S1

**mRNA analysis of intestinal mucosal biopsies assessed by NanoString technology**

| 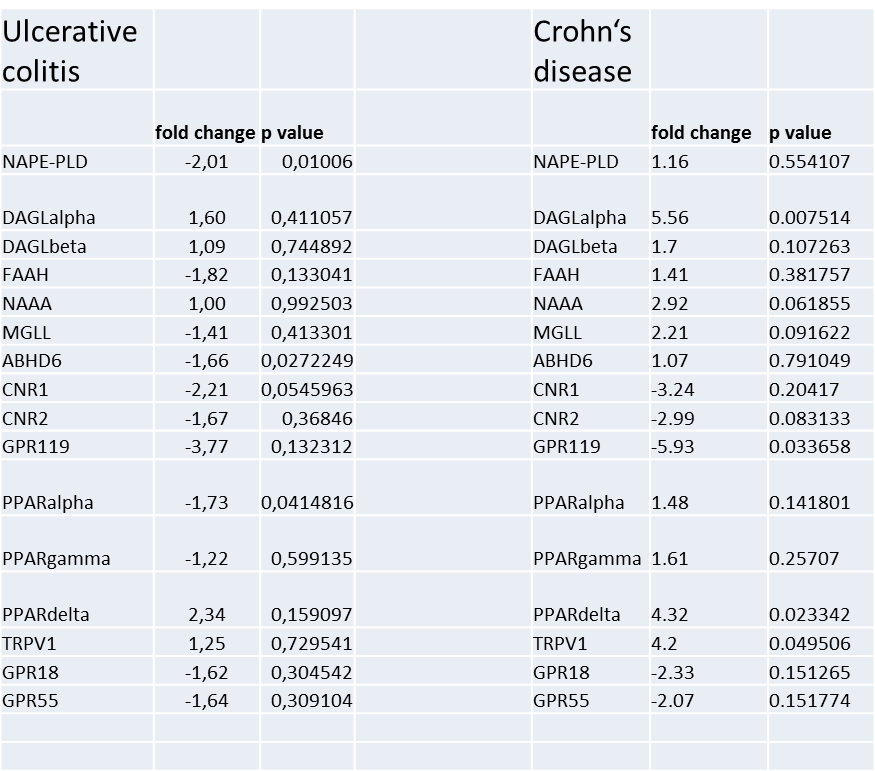 |  |  |  |  |  |  |
| --- | --- | --- | --- | --- | --- | --- |

Supplementary Table S2

**Mass spectrometry**

**Information on gradient and flow rate.**

| **Time** | **Flow rate (µL/min)** | **Solvent A (%)** | **Solvent B (%)** |
| --- | --- | --- | --- |
| 0.00 | 500 | 80.0 | 20.0 |
| 0.50 | 500 | 80.0 | 20.0 |
| 0.60 | 500 | 40.0 | 60.0 |
| 8.00 | 500 | 36.5 | 63.5 |
| 8.01 | 500 | 5.0 | 95.0 |
| 10.00 | 500 | 5.0 | 95.0 |
| 10.01 | 500 | 80.0 | 20.0 |
| 12.00 | 500 | 80.0 | 20.0 |

**Information on LC- and MS-parameter.**

| **parameter** | **setting** |
| --- | --- |
| Injection volume | 10 µL |
| Column oven temperature | 50 °C |
| Curtain gas (nitrogen) | 45 psi |
| Ionspray voltage | 4500 V |
| Source temperature | 450 °C |
| Nebulizer gas (nitrogen) | 40 psi |
| Auxiliary/Turbo heater gas (nitrogen) | 60psi |
| Collision gas (nitrogen) | 9 psi |
| Detection MS/MS | Multiple Reaction Monitoring (MRM) |
| Q1/Q3 resolution | unit/unit |

**Information on MRM-transitions, used IS and LLOQ- and ULOQ-values for all analytes.**

| **Analyt** | **Q1** | **Q3 (Quan/Qual)** | **IS** | **LLOQ (ng/mL)** | **ULOQ (ng/mL)** |
| --- | --- | --- | --- | --- | --- |
| AEA | 348.3 | 287.3 / 62.2 | AEA-d_8_ | 0.10 | 2.00 |
| PEA | 300.2 | 62.2 / 283.2 | PEA-d_4_ | 0.50 | 10.00 |
| OEA | 326.3 | 62.2 / 309.2 | OEA-d_4_ | 0.25 | 5.00 |
| 1-AG* | 379.2 | 287.3 / 269.2 | 1-AG-d_5_* | 0.25 | 5.00 |
| 2-AG* | 379.2 | 287.3 / 269.2 | 2-AG-d_5_* | 0.25 | 5.00 |

*1-AG and 2-AG cannot be distinguished by mass spectrometric detection but were base line separated by the used LC-method.

Supplementary information S3

**LPI extraction and measurement**

Lipid extraction was carried out from 150 μL of plasma sample in the presence of 10 μL of LPI 17:1 (100 μM) as internal standard according to a modified version of the original extraction protocol published by Matyash et al. (2008). Methanol (1.5 mL) and MTBE (5 mL) were added to the samples in 12 mL glass tubes with teflon lined caps and the mixture was incubated for 10 min in an overhead shaker at room temperature. After addition of 1.25 mL deionized water and 10 min of additional shaking, the mixture was centrifuged for 5 min at 1350 g and the upper phase was transferred to a new glass tube. The lower phase was re-extracted with 2 mL of the upper phase of MTBE/methanol/deionized water (10:3:2.5, v/v/v) and again the upper phase was collected, combined with the upper phase from the first extraction, evaporated in a vacuum centrifuge (Thermo Fisher Scientific, Waltham, MA, USA) and dissolved in 500 µL chloroform/methanol (1:1, v/v) for storage at −80 °C. Prior to analysis, the storage solvent was evaporated under a gentle stream of nitrogen and the sample reconstituted in the same volume of injection solvent isopropanol/ chloroform/ methanol (90:5:5, v/v/v).

LC-MS measurements for LPI quantification were performed with slight modifications as previously described in Triebl et al. (2017). Briefly, chromatographic separation was performed on a Waters (Waters, Milford, MA, USA) BEH C8 column (100 × 1 mm, 1.7 µm), thermostatted to 50 °C in a Dionex Ultimate 3000 RS UHPLC system. Mobile phase A was deionized water containing 1 vol% of 1 M aqueous ammonium formate (final concentration 10 mmol/L) and 0.1 vol% of formic acid as additives. Mobile Phase B was a mixture of acetonitrile/isopropanol 5:2 (v/v) with the same additives. Gradient elution started at 50% mobile phase B, rising to 100% B over 15 min; 100% B were held for 10 min and the column was re-equilibrated with 50% B for 8 min before the next injection. The flow rate was 150 µL/min, the samples were kept at 8 °C and the injection volume was 2 µL. The Orbitrap Velos Pro hybrid mass spectrometer (Thermo Fisher Scientific Inc., Waltham, MA, USA) was operated in Data Dependent Acquisition mode using an inclusion list of all theoretical possible LPI species. Prior to experiments, lens settings were tuned and source parameters were optimized to the signal of LPI 17:1 using the deprotonated molecule. Every sample was measured in negative polarity using a HESI II ion source. Ion source parameters for negative polarity were as follows: Source Voltage: 3.9 kV; Source Temperature: 400 °C; Sheath Gas: 35 arbitrary units; Aux Gas: 10 arbitrary units; Sweep Gas: 0 arbitrary units; Capillary Temperature: 300 °C. Automatic gain control target value was set to 10^6^ ions to enter the mass analyzer, with a maximum ion accumulation time of 500 ms. Full scan profile spectra from m/z 425 to 900 in negative ion mode were acquired in the Orbitrap mass analyzer at a resolution setting of 100 000 at m/z 400. For MS/MS experiments, the 10 most abundant ions of the full scan spectrum - using a inclusion list of deprotonated LPI species - were sequentially fragmented in the ion trap using He as collision gas (CID, Normalized Collision Energy: 50; Isolation Width: 1.5; Activation Q: 0.2; Activation Time: 10) and centroided product spectra at normal scan rate (33 kDa/s) were collected. The exclusion time was set to 11 s.

LC/MS data were processed using Lipid Data Analyzer (LDA) (Hartler et al., 2017)). Briefly, the algorithm identifies lipids with a 3D algorithm, using the three dimensions m/z, retention time, and intensity to correctly integrate peaks, while also taking into account the isotopic distribution. MS/MS spectra are considered for confirmation of structures by characteristic head group and fatty acyl fragments. Lipids are annotated according to the official international shorthand nomenclature (Liebisch et al., 2013).

Hartler J, Triebl A, Ziegl A, Trötzmüller M, Rechberger GN, Zeleznik OA, Zierler KA, Torta F, Cazenave-Gassiot A, Wenk MR, Fauland A, Wheelock CE, Armando AM, Quehenberger O, Zhang Q, Wakelam MJO, Haemmerle G, Spener F, Köfeler HC, Thallinger GG. Deciphering lipid structures based on platform-independent decision rules. *Nat Methods*. 2017;14(12):1171-1174. doi: 10.1038/nmeth.4470.

Liebisch G, Vizcaíno JA, Köfeler H, Trötzmüller M, Griffiths WJ, Schmitz G, Spener F, Wakelam MJ. Shorthand notation for lipid structures derived from mass spectrometry. *J Lipid Res*. 2013;54(6):1523-30.

Matyash V, Liebisch G, Kurzchalia TV, Shevchenko A, Schwudke D. Lipid extraction by methyl-tert-butyl ether for high-throughput lipidomics. *J Lipid Res*. 2008;49(5):1137-46. doi: 10.1194/jlr.D700041-JLR200.

Triebl A, Trötzmüller M, Hartler J, Stojakovic T, Köfeler HC. Lipidomics by ultrahigh performance liquid chromatography-high resolution mass spectrometry and its application to complex biological samples.

*J Chromatogr B Analyt Technol Biomed Life Sci*. 2017;1053:72-80. doi: 10.1016/j.jchromb.2017.03.027.
